# Osteoarthritis: Novel Molecular Mechanisms Increase Our Understanding of the Disease Pathology

**DOI:** 10.3390/jcm10091938

**Published:** 2021-04-30

**Authors:** Susanne Grässel, Frank Zaucke, Henning Madry

**Affiliations:** 1 Department of Orthopaedic Surgery, Experimental Orthopaedics, Centre for Medical Biotechnology (ZMB), Bio Park 1, University of Regensburg, 93053 Regensburg, Germany; 2 Dr. Rolf M. Schwiete Research Unit for Osteoarthritis, Orthopedic University Hospital Friedrichsheim, 60528 Frankfurt am Main, Germany; frank.zaucke@kgu.de; 3 Center of Experimental Orthopaedics, Saarland University, 66421 Homburg, Germany; henning.madry@uks.eu

**Keywords:** OA, cartilage, synovitis, subchondral bone, inflammation, neuropeptides, macrophages, cytokines, topographical pattern, joint innervation

## Abstract

Although osteoarthritis (OA) is the most common musculoskeletal condition that causes significant health and social problems worldwide, its exact etiology is still unclear. With an aging and increasingly obese population, OA is becoming even more prevalent than in previous decades. Up to 35% of the world’s population over 60 years of age suffers from symptomatic (painful, disabling) OA. The disease poses a tremendous economic burden on the health-care system and society for diagnosis, treatment, sick leave, rehabilitation, and early retirement. Most patients also experience sleep disturbances, reduced capability for exercising, lifting, and walking and are less capable of working, and maintaining an independent lifestyle. For patients, the major problem is disability, resulting from joint tissue destruction and pain. So far, there is no therapy available that effectively arrests structural deterioration of cartilage and bone or is able to successfully reverse any of the existing structural defects. Here, we elucidate novel concepts and hypotheses regarding disease progression and pathology, which are relevant for understanding underlying the molecular mechanisms as a prerequisite for future therapeutic approaches. Emphasis is placed on topographical modeling of the disease, the role of proteases and cytokines in OA, and the impact of the peripheral nervous system and its neuropeptides.

## 1. Introduction

More than 10% of the world population show clinical symptoms of osteoarthritis (OA), affecting most individuals above the age of 65. The population of the European community belongs to the most long-living worldwide, and is increasingly suffering from obesity. As a consequence, the economic and social burden caused by OA is growing rapidly, and they substantially influence the life quality of affected individuals. This development implements enormous costs to the health care systems for diagnosis, treatment, sick leave, rehabilitation, and early retirement. Moreover, OA is often linked to chronic pain, which prevents those afflicted from participating in social activities, working and maintaining an independent lifestyle [1]. OA emanates from the dysfunction of the whole joint, especially affecting the articular cartilage, synovium and subchondral bone, and tissues with close mechanical and molecular biological interactions [2,3,4]. In addition, OA is characterized by a strong hereditary background [5]. Variant alleles of several genes, often strongly expressed in cartilage, mostly lead to increased risk of developing hip or knee OA. For example, a functional evaluation of Growth Differentiation Factor (GDF) 5, identified the gene variant rs6060369 as causative for an increased risk for developing OA due to aging [6]. Functional data demonstrating the upregulation of GDF5 expression in OA cartilage during cartilage repair and as a response to an inflamed and injured synovium in mice supported this association [7]. 

The development of targeted therapies against the osteoarthritic processes in cartilage, synovium or subchondral bone will, therefore, require an understanding of the state of these joint tissues at the time of the intervention. Importantly, interventions will mostly not be successful unless they are applied at the early stages of the disease before considerable structural and functional alterations occur in the osteochondral unit. 

The structural patterns of osteochondral changes that reflect the beginning of the disease are incompletely understood. Specific topographical patterns of early articular cartilage and subchondral bone changes are observed after defined OA induction in large animal models resembling phenotypes, as seen in patients [8]. These patterns arise locally and progress globally, precisely indicating disease progression, as they progressively disturb the strong tissue connections existing within a normal osteochondral unit. 

OA is also associated with a variable degree of synovial inflammation, which may play a significant role particularly in early OA [9]. Inflammatory processes include the presence of high levels of plasma proteins, complement components, proteases and cytokines in the synovial fluid and other joint tissues. Critical players are immune cells, such as macrophages and lymphocytes, immigrated and activated in the synovial membrane and fluid, which trigger catabolic and inflammatory cellular responses. At the molecular level, these processes are regulated by a complex network of proteolytic enzymes, chemokines, cytokines and neuropeptides.

Joint tissues such as the synovium, articular cartilage, meniscus and subchondral bone, including their cellular components, are targets for neuropeptides. In particular, sensory neuropeptides play a pivotal role in inflammatory processes, together with aberrant pro-inflammatory cytokine levels and reduced regenerative signaling factors. Changes in peripheral joint innervation and neuropeptide supply are supposed to be partly responsible for degenerative alterations in joint tissues, which contribute to the development of OA [10]. Altogether, it is evident that sensory neurotransmitters have crucial trophic effects, which are critical for joint tissue function and bone homeostasis. They modulate articular cartilage, subchondral bone and synovial tissue properties in physiological and pathophysiological conditions in addition to their classical neurological features. A better understanding of changes in the sensory neuropeptide supply might lead to novel strategies to halt OA progression.

Choosing appropriate therapies/medication for selective disease time-points and subgroups of patients might help to tailor individual treatment regimens for each patient in the future. It is crucial to define the biomechanical and biochemical mechanisms and pathways involved in the crosstalk between the different joint tissues in order to identify novel targets and develop therapies to improve the quality of life and restore the ability to work for OA patients. This review elaborates on the mechanisms responsible for structural changes in joint tissues at the very early stage of OA. Furthermore, catabolic responses of articular cartilage to an increased pro-inflammatory environment that induces cartilage matrix breakdown, will be discussed. The final part elaborates on the contribution and role of sensory neuropeptides in joint tissue pathology and their involvement in modulating inflammatory processes in the joint during OA pathogenesis. 

## 2. Novel Aspects of Early Topographic Modeling of Human OA

### 2.1. Structural Changes in Human Early OA

The early stage of OA affects not only the articular cartilage and the subchondral bone, but also the menisci, the synovial membrane, the joint capsule, ligaments and muscles [2,11]. Early OA is an important period during OA progression [12], since alterations at this stage may potentially be reversible [13] compared to the late stages, when most cartilage is either lost or severely pathologically altered [14]. However, the structural patterns of osteochondral changes reflecting the onset of the disease are only incompletely understood [15]. Emerging data from the past decades indicate that OA, especially knee OA, may be caused by specific pathological insults that, once they take place, initiate the breakdown of the superficial articular cartilage and lead to changes in the subchondral bone [2]. 

The histopathological features of OA are especially important and ideally suited for illustrating the changes in the early OA phase [16,17,18]. In the beginning, a superficial articular cartilage loss occurs, which over time will affect and abrade the entire cartilage, often down to the subchondral bone [19]. Based on the linear grading and staging system proposed by the Osteoarthritis Research Society International (OARSI), an intact articular cartilage surface would receive an OARSI grade of 1.0 [20]. The very early histopathological features of OA are considered to represent a hypertrophic repair attempt of the articular cartilage [16]. In this phase, the thickness of the cartilage increases. This swelling probably results from the damage to the collagen network, which leads to an increase in its water content. In this anabolic phase, compensatory mechanisms occur, among which an increased synthesis of cartilage extracellular matrix (ECM) molecules (proteoglycans and type II collagen) by the proliferating articular chondrocytes that form clusters is observed [16]. The spatial organization of the superficial chondrocytes is, therefore, disturbed in early OA, even before macroscopic changes become visible [21]. 

Probably at a point when the damaged articular cartilage is not capable anymore of counterbalancing the pathological forces, catabolic activities reverse this early anabolic phase. Here, an amplified synthesis of matrix metalloproteinases (MMPs), aggrecanases (ADAMTS-4 and ADAMTS-5), regulatory proteins, stress and apoptotic markers as well as transcription factors occurs [22,23]. They induce the loss of the superficial cartilage zone, and fibrillations of this cartilage zone occur. This first phase of discontinuity reflects OARSI grade 2.0. Abrasion of the surface together with the proteoglycan loss in the superficial zone represents OARSI grade 2.5. The superficial ECM loss is followed by enlarged fibrillations, becoming simple and then complex fissures. When vertical fissures extend into the mid zone of the cartilage, OARSI grade 3.0 is reached. The next stages are not considered to reflect early OA anymore, and consequently, the thickness of the articular cartilage progressively decreases [24].

Changes during early OA also occur in the subchondral bone, the composite formed by the subchondral bone plate and the subarticular spongiosa [25,26]. The turnover [27] and thickness of the subchondral bone plate progressively increases, osteophytes form at the margins of the joint, and the subarticular spongiosa is remodeled [4]. A novel histological scoring system for subchondral bone changes in murine models of joint aging and OA was recently published [28]. In early phases of OA, the changes of the subchondral bone start with an undulation of the subchondral bone—cartilage interface, followed by a slight, and over time, considerable, increase in subchondral bone plate thickness and volume. Angiogenesis within the subchondral bone plate also occurs [26], possibly in conjunction with the remodeling process of the subchondral bone plate that involves both osteoclasts and osteoblasts [4]. Osteophytes also occur in early OA [29]. Once developed, their size may increase, while no correlation between the regional distribution of osteophytes and the region affected by OA exists [8]. In later stages that are not considered early OA anymore, osteoclast activity extends into the calcified cartilage, vascular ingrowth into the articular cartilage occurs and osteoblasts infiltrate, which deposit novel bone that results in end-stage OA sclerosis [28]. 

### 2.2. What Initiates the Breakdown of the Osteochondral Unit?

Risk factors initiating the breakdown of the osteochondral unit are various, and their detailed discussion is outside the scope of this article. However, they all lead to an imbalance of anabolic and catabolic forces, which ultimately disturbs this critical balance. While OA was classically separated into primary OA, based on a genetic predisposition, and secondary OA, caused by different factors, it became clear that the risk factors for OA development are patient age-specific and overlapping [30]. Developmental dysplasia of the hip is a pre-osteoarthritic deformity that occurs in infants and represents a serious and leading cause of OA at an early age [31]. Traumatic meniscal tears [32], rupture of the anterior cruciate ligaments [33], focal (osteo) chondral defects [34] and direct trauma to the cartilage [35] may occur at all ages, and all play a role in the onset of OA [3,36]. They are exacerbated by overload to the affected joint, for example, caused by obesity [37] or axial malalignment [38]. 

Varus malalignment of the lower extremity increases the risk of incident medial cartilage damage [39], and OA onset [40] and progression [41]. The degree of increase in malalignment is significantly associated with cartilage thickness loss in OA [42], although earlier reports did not link alignment with OA incidence [43]. Data from the Multicenter Osteoarthritis Study (MOST) and the Osteoarthritis Initiative (OAI) showed that also the risk of lateral knee OA incidence and radiographic progression is increased in valgus malalignment [44]. In advanced human varus knee OA, the overloaded and severely damaged medial tibial plateau becomes unresponsive [38]. Interestingly, in the lesser loaded (lateral) site, the degree of malalignment correlates negatively with cartilage degeneration and subchondral bone plate thickening, thus revealing a site-specific effect of varus malalignment. These findings underscore the need to determine axial alignment in clinical trials while also highlighting the importance of determining the correct compartment and OA grade when assessing potential therapeutic tissue changes [45]. 

### 2.3. Meniscal Tears Lead to OA

Menisci and cartilage form a functional unit, and lesions of the menisci are among the most important known causes of knee OA. In a normal knee, 50% of the load in the medial compartment is transmitted through the menisci [46] in a well-distributed fashion, as long as the menisci are intact [47]. Menisci also absorb intermittent shock waves generated by a normal gait, which decrease by 20% in meniscectomized knees [46]. While the geometric structure of the menisci supports joint congruity and stability, removal of the medial meniscus results in a 100% increase in contact stress ex vivo, significantly increasing load, which may induce articular cartilage damage and degeneration [46,48]. Consequently, meniscal damage is strongly associated with the onset and progression of tibiofemoral OA. Crema and co-workers identified an association between prevalent medial meniscal pathology and medial compartmental cartilage loss over a 2-year period in 152 women without or with knee OA [49]. Bloeker and co-workers studied whether medial meniscal extrusion in patients is more strongly associated with cartilage loss in certain medial tibio-femoral subregions than in others [50]. Their data showed that in OA patients, the submeniscal medial tibial cartilage is particularly affected by the reduced meniscal coverage resulting from medial meniscus extrusion. Evidence of a specific macroscopic pattern of early OA and strong correlation with meniscal damage further supported this location-dependent concept [51]. Patients with a symptomatic isolated meniscal defect with otherwise stable knees displayed, already, early radiographic knee OA in the medial tibiofemoral compartment as identified by arthroscopic scoring of meniscal and articular cartilage damage and Kellgren-Lawrence (KL) grading. A significant correlation between the localizations of the meniscal tissue loss and the early OA cartilage lesions existed [8]. Most lesions were seen in the posterior meniscal horn and posterior third of the medial tibial plateau cartilage, most likely because the human tibial plateau slides back during knee flexion over the femoral condyles, prompting higher compressive loads.

### 2.4. Topographic Modeling of Human OA

Since small animal models enable only limited insights because of their size, studies in large animals are of value to validate such topographical changes seen in clinical studies. The induction of knee OA in adult sheep through a defined partial anterior medial meniscectomy (pMMx) allowed to model at high resolution the topographic pattern of early OA affecting the osteochondral unit [8]. KL grading of standard radiographs at 6 weeks following pMMx resembled early radiographic OA that was worse but not significantly different compared to normal controls, a finding echoing the clinical challenge of identifying early OA changes on standard radiographs [52]. In the ovine model, the pattern of macroscopic and microscopic early OA changes started in the specific (anterior) part of the tibial plateau below the region affected by the meniscus loss. Specific histopathological findings reflecting early OA included fissures, fibrillations and cartilage erosions, reduced chondrocyte density and a disturbed architecture of the superficial cartilage layer. Likewise, osteophyte formation was exclusively traced to the periphery of the anterior and intermediate regions of the medial tibial plateaus. Differential alterations within the subchondral bone were also found. 

**Figure 1:**
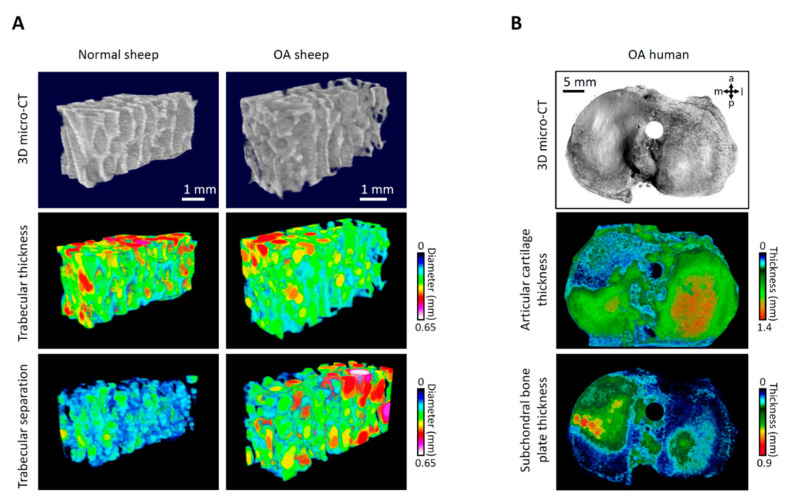
Topographic pattern of osteoarthritis (OA) in sheep and human tibial plateaus. (**A**) Representative 3-dimensional (3D) microcomputed-tomography (micro-CT) reconstruction and color-coded 3D models of trabecular thickness (Tb.Th) and separation (Tb.Sp) of the subarticular spongiosa of the anterior medial tibial plateau of a normal and an osteoarthritic sheep 6 weeks after OA induction by anterior partial meniscectomy. (**B**) Structural overview and color-coded 3D micro-CT models of articular cartilage and subchondral bone plate thickness of a representative human tibial plateau.

Although a thickening of the subchondral bone plate was identified, interesting data emerged from the subarticular spongiosa. Exactly below the region of the initial articular cartilage damage induced by pMMx, the subarticular spongiosa developed initial changes, which then extended and led to a deterioration of the trabeculae of the subarticular spongiosa in the entire medial tibial plateau [8]. These strong topographical correlations between cartilage degeneration, thickness of the subchondral bone plate, and different microstructural parameters of the subarticular spongiosa (Figure 1) support the clinical concept of a location-dependent development of OA. Data on structural knee OA progression from clinical trials exposed longitudinal geographical changes in articular cartilage thickness that are highly depending on the evaluated subregion. Such location-independent analyses (in contrast to the “static” change in radiographic joint space width in the medial femorotibial compartment, as requested by regulatory bodies) may serve as novel endpoints of clinical trials, especially when examining anabolic therapies [51].

## 3. The Role of Proteases and Cytokines in OA

### 3.1. Proteases in OA

The degeneration but also the remodeling of the different tissues in the joint during progression of OA is highly dependent on the activity of a large number of different proteases. This review will mainly focus on the degradation of the cartilage extracellular matrix (ECM), as the loss of proteoglycans from the articular cartilage surface is one of the earliest signs and major histological hallmarks of OA. Further, the molecular changes in this area have been investigated in numerous studies and are much better characterized than in other tissues. Articular cartilage contains two superstructures, a collagen meshwork and a proteoglycan gel [53], providing the tissue with both tensile strength and compressive resistance, respectively. The main collagen type in cartilage is collagen II, which forms first fibrils and then networks that are further stabilized by other minor collagen types (e.g., VI, IX, XI) and non-collagenous proteins, such as matrilins, thrombospondins and many others. These proteins, together with small leucine rich proteoglycans, including decorin and biglycan, connect the collagen network with aggrecan, the major proteoglycan in cartilage [54]. As all these proteins are mainly found in the periphery of collagen fibrils, they are also referred to as perifibrillar proteins [55]. 

During the progression of OA, the initial and continuous loss of proteoglycans from the ECM is accompanied by the degradation of quantitatively minor ECM components before the degradation of the collagen fibrillar network takes place [56,57]. The major cartilage degrading enzymes belong to the families of MMPs and a disintegrin and metalloproteinase with thrombospondin-like motifs (ADAMTS). MMPs are zinc-dependent endopeptidases also referred to as matrixins. The 28 members of the MMP family differ structurally as well as in their tissue expression and substrate specificity [58]. The collagenases MMP-1, -3, -9 and -13 are considered to play the most important role in OA; the key role in collagen II degradation has been assigned to MMP-13. Even though collagen II degradation takes place within the cartilage ECM, MMP-13 is not only expressed by chondrocytes but also secreted by osteocytes and synovial fibroblasts, which might, in that way, contribute substantially to cartilage degeneration. Nevertheless, the term collagenase might be misleading, as MMP-13 and others are also able to cleave non-collagenous ECM components; some of them even digest aggrecan. However, it has been demonstrated that MMPs are less efficient in aggrecan cleavage compared to ADAMTS-4 and -5, consequently also referred to as aggrecanase-1 and -2 [59]. 

Based on the central role of MMPs in cartilage degeneration, it was attractive to speculate that broad-spectrum MMP inhibitors might be used for treatment of OA. However, clinical trials did not show a clear benefit in patients with knee OA after administration of such an inhibitor but severe side effects in the musculoskeletal system, making them unsuitable for use in OA therapy [60,61]. Further studies are underway to develop selective MMP-13 inhibitors or even dual inhibitors of MMP-13 and aggrecanases [62].

Both aggrecanases, ADAMTS-4 and -5, are expressed in joint tissues. However, the exact contribution of these two enzymes to cartilage degeneration in OA was unclear for a long time. Only when mouse lines deficient in individual aggrecanases were generated and characterized, it turned out that ADAMTS-5 is the major aggrecanase in murine cartilage [63]. Very recently, another mouse model indirectly confirmed the crucial role of ADAMTS-5. Overexpression of the aggrecanase-selective tissue inhibitor of metalloproteinase-3 (TIMP-3) protected articular cartilage in a surgical murine OA model [64]. This identification of ADAMTS-5 as the key aggrecanase in both animal models and human explant cultures encouraged the development of specific ADAMTS-5 inhibitors. The company Galapagos developed a monoclonal antibody as a highly selective ADAMTS-5 inhibitor (GLPG1972) and validated it successfully in a rat meniscectomy model [65]. In a phase 1b trial in OA patients, GLPG1972 reduced the serum concentration of aggrecan degradation products by over 50% over a four-week period. Currently, this inhibitor is in a phase 2b trial (Roccella trial, NCT03595618) with knee OA patients, but the outcome of this trial has not yet been published.

### 3.2. Cartilage ECM Protein Fragments

The degradation of the cartilage ECM by MMPs and ADAMTSs leads, obviously, to a loss of structural support and mechanical properties of the tissue, but at the same time to the liberation of cleavage products that are released into the synovial fluid. Proteins and, in particular, smaller fragments thereof finally appear in the serum where they can be easily detected and thus be used as biomarkers for the state of degeneration. One widely used example is the C-telopeptide of collagen II (CTX-II) as a marker for the progression of cartilaginous lesions. Its serum concentration directly correlates with radiological grades and clinical scores for OA. Recently, its diagnostic performance has been reviewed systematically [66]. The serum concentration of the cartilage oligomeric matrix protein (COMP), a collagen-binding protein that is involved in the secretion of collagen molecules [67] and their arrangement in fibrils and networks [68], correlates with both disease severity and the number of affected joints [69]. However, over the last years, a number of studies have shown that serum COMP also increases in fibrotic diseases and various forms of cancer [70], questioning its validity as a specific biomarker. Currently, the potential of so-called neoepitope antibodies that would allow detection of OA-specific cleavage products of COMP in synovial fluid is under investigation [71,72].

The cartilage component lubricin that is almost exclusively expressed in the superficial layer of articular cartilage has been shown to be efficiently degraded by cathepsin G. A specific 25 kDa degradation fragment was detected in the synovial fluid of OA patients [73], and its potential to serve as a biomarker is currently under investigation. In search of both predictive and descriptive OA biomarkers, other cartilage ECM components were identified in bioinformatic approaches. Co-expression network and pathway analysis of gene expression profiles from different OA datasets were performed [74,75]. The expression profile of genes clearly associated with OA (like e.g., MMP2, COL6A1, COL9A1-3) and the synthesis and degradation of the encoded proteins has to be evaluated in future studies.

Interestingly, the fact that cartilage ECM components and cleavage products might stimulate the immune system or even exert biological functions in the joint has only been recognized in recent years. Indeed, several studies have shown that antibodies directed against cartilage matrix proteins or fragments can be detected in serum samples of patients suffering from OA [76] and RA [77]. In a recent elegant study, a functional coupling between such autoantibodies and pain has been demonstrated. Antibodies specific for collagen II or COMP elicited a mechanical hypersensitivity in mice, uncoupled from visual, histological and molecular indications of inflammation. In this case, pain was induced through the immune-complex-mediated activation of neurons [77].

Several examples indicate that cleavage products might play an active role in different processes of OA pathology: the continuous cleavage of aggrecan by the joint action of MMPs and ADAMTSs eventually results in the generation of a 32-amino acid-comprising fragment [78]. This fragment has been shown to drive OA pain via toll-like receptor (TLR)-2 and a dose-dependent release of CCL2 [79]. Treatment of primary human chondrocytes with fragments of collagen II as well as the collagen associated protein matrilin-3 induced a concentration- and time-dependent release of interleukin (IL)-1, -6, -8, TNFα and MMP-1, -3, -13 [80]. Interestingly, larger fragments of COMP have not been able to do so in identical in vitro experiments [81]. Very recently, the effect of smaller COMP-derived peptides that have been detected in the degenerated cartilage tissue of OA patients was studied in different in vitro assays. Here, COMP peptides turned out to be rather inert and did not affect the viability of osteo-chondroprogenitor cells, nor the tube formation capacity of endothelial cells nor the cytokine release in synovial explant cultures [82]. 

However, there are also examples of matrix proteins and fragments that have clear beneficial effects that might be relevant for OA pathology. The cartilage matrix glycoprotein tenascin C was shown to control the maturation of articular cartilage in mice [83]. Interestingly, the intraarticular injection of this protein decelerated the progression of surgically induced OA in a murine model [84]. A follow-up study identified TNIIIA2, a 22-mer peptide of tenascin C, as the active part of the molecule that prevented cartilage degeneration as well as synovitis [85]. Specific fragments of fibronectin, another glycoprotein highly abundant in a variety of different ECMs, were recently shown to support stem cell chondrogenesis [86], a process that might also be relevant in the regeneration of articular cartilage. Link protein is a glycoprotein that interacts with and connects hyaluronic acid and aggrecan, and thus stabilizes the cartilage ECM. Recent studies have demonstrated that a short N-terminal fragment has anabolic functions, promotes matrix production and decreases the activity of catabolic enzymes, including MMPs, in degenerating intervertebral discs [87] but also human OA cartilage [88]. The fragmentation of the perifibrillar proteoglycans decorin, biglycan, lumican and keratocan is elevated in degenerating human meniscus, knee and hip articular cartilages compared with age-matched macroscopically normal and control tissues [89]. However, the function of their degradation products has not yet been analyzed systematically. The number of bioactive degradation products will surely increase in the near future and unravelling their mode of action on different cell types in the joint might contribute to a better understanding of mechanisms in OA pathology.

### 3.3. Cytokines in OA

It is well accepted that OA has an inflammatory component and that the concentration of pro-inflammatory mediators, including cytokines, is increased in animal models and patients with OA. Over the last decades, it has been shown in numerous in vitro studies that ECM fragments have a multifaceted impact on inflammatory cells. ECM-derived fragments exhibit chemotactic activity for inflammatory cells, enhance phagocytic functions, induce immune responses and changes in gene expression as well as the release of cytokines by inflammatory cells [90]. Pro-inflammatory cytokines are also critical mediators in the disturbed metabolism and enhanced catabolism of the different tissues in the OA joint [91]. The main cytokines involved in the pathophysiology of OA seem to be IL-1β, tumor necrosis factor (TNF), IL-6 and, to a minor extent, IL-15, IL-17 and IL-18 [92]. These cytokines can be released by inflammatory cells, but, as mentioned above, there is considerable in vitro evidence that fragments or peptides derived from cartilage ECM components induce the release of these cytokines also in cells of an articular joint, including chondrocytes, bone and synovial cells [9]. 

IL-1β is considered a key cytokine in OA for several reasons. In OA patients, its concentration is increased in various tissues of the joint [93]. At the same time, the expression of the corresponding receptor IL-1R1 is elevated in chondrocytes and synovial fibroblasts [94,95]. Further, IL-1β inhibits the synthesis of the major cartilage ECM components collagen type II and aggrecan and does induce the expression of matrix-degrading proteases of the MMP and ADAMTS families [9]. Anti-cytokine therapies targeting IL-1β production or activity have also been considered for treating human OA [96]. Treatment strategies include the modification or inhibition of IL-1β action through the application of IL-1β receptor antagonists, soluble IL-1β receptors, monoclonal antibodies against IL-1β or against its receptor, blocking the formation of active IL-1β or blocking the IL-1 dependent intracellular signaling pathways. These strategies have been investigated in numerous preclinical and clinical studies [97]. However, despite some promising effects in these studies, clinical translation remains a challenge and further research is needed to achieve the desired therapy outcome [98]. Very similar inhibitory effects on matrix protein synthesis as well as the stimulation of matrix degrading enzymes have been reported for TNF-α [99,100]. More recently, animal studies have provided additional evidence that blocking TNF-α and IL-1β production could counteract the degradative mechanisms associated with OA pathology [101,102]. Similar to IL-1β and TNF-α, IL-6 is elevated in both serum and synovial fluid (SF) of OA patients and is a potent inducer of MMPs [103]. In mice subjected to DMM surgery, IL-6 induced cartilage degeneration via Stat3 signaling, and the systemic inhibition of this pathway was protective against experimental OA [104]. A clinical trial using an IL-6 receptor-neutralizing antibody (tocilizumab) in patients with hand OA was completed in 2019 and the results were published very recently, showing that, at least for pain relief, this treatment in patients was no more effective than placebo [105].

In addition to classical cytokines, a large number of other small proteins and growth factors can play a role in OA. Chemokines can be regarded as a subfamily of cytokines with low molecular weight. Depending on the position of their cysteine (C) residues, they are classified into four families: CXC, CC, C, CXC3. The best studied chemokine in OA is the CC motif ligand 2 (CCL2), also referred to as MCP1 (monocyte attractant protein 1). Serum levels and the concentration of CCL2 in the SF of OA patients are elevated [106] and single nucleotide polymorphisms in the *CCL2* gene have been associated with the risk of developing OA [107]. As reported for the abovementioned cytokines, stimulation of chondrocytes with CCL2 leads to decreased matrix protein synthesis but increased MMP expression [108]. CCL2-deficient mice were protected against OA with a concomitant reduction in local monocyte/macrophage numbers in their joints [106]. Almost all members of the different chemokine families induce a catabolic and pro-inflammatory response in chondrocytes. One exception is the CXCR2 receptor and its ligand CXCL6 that seem to have an important role in cartilage homeostasis and the maintenance of a healthy chondrocyte phenotype [109]. 

CCN proteins comprise another family of ECM-associated proteins involved in intercellular signaling, with relevance in OA. As dynamically-expressed non-structural ECM proteins, they are also referred to as matricellular proteins [110]. The CCN family consists of six members, including CCN1-6, which correspond to cysteine-rich protein 61 (Cyr61), connective tissue growth factor (Ctgf), nephroblastoma overexpressed (Nov), Wisp1, Wisp2, and Wisp3, respectively. The expression of all six CCN genes was found to be increased in OA and RA (rheumatoid arthritis) synovial samples and knee cartilage as compared to healthy controls [111]. The contribution of the individual CCN proteins has been studied in more detail. CCN1 has been shown to aggravate cartilage inflammaging and matrix degradation. In addition, overexpression of CCN1 promoted chondrocyte senescence [112]. Another study revealed that CCN1 suppresses ADAMTS-4 activity but is directly associated with chondrocyte cloning and cluster formation in OA cartilage [113]. In addition, CCN4 exacerbated cartilage degeneration in experimental OA [114], while CCN2 and CCN3 have cartilage protective or even regenerative effects [115,116].

In addition to these chondroprotective members of the CCN family, there are a couple of other pro-anabolic factors with relevance to OA. Their effects in inhibiting matrix degradation, promoting chondrogenesis and reducing inflammation make them promising candidates for OA therapies. Among those are the bone morphogenetic protein (BMP)-7, the insulin-like growth factor (IGF)-1 and the fibroblast growth factor (FGF)-18. Their potential has been demonstrated in several cell and tissue culture or animal models [117,118]. Sprifermin, which is a truncated version of human FGF18, has been shown to induce chondrocyte proliferation and cartilage matrix production [119,120]. Respective promising clinical trials are currently ongoing. One of them represents a phase II multicenter randomized dose-finding clinical study [121]; clinicaltrials.gov NCT01919164), investigating the intra-articular administration of sprifermin. A meta-analysis of the efficacy and safety of sprifermin injection for knee OA treatment has been published very recently [122]. In parallel, a large number of other cytokine/growth factor therapies are in different phases of clinical trials and it will be interesting to see if any of these treatments will be effective in OA therapy [123,124]. Finally, a number of recent studies suggest that the Wnt-signaling pathway might act as a key regulator and activator of molecular and cellular processes during OA development [125]. This pathway modulates the differentiation of chondrocytes and osteoblasts as well as the synthesis of catabolic proteases. Therefore, lorecivivint (SM04690), a small-molecule Wnt pathway inhibitor, was evaluated in a series of in vitro and in vivo animal studies to determine its effects on OA pathology [126,127]. In a 24-week, randomized, controlled phase 1 study, this inhibitor appeared to be safe and well tolerated, and positive trends for disease-modifying osteoarthritis drug (DMOAD) properties were suggested [128]. The first promising results of a phase II randomized trial were published in 2020 [129]. Even though this trial did not meet its primary endpoint, preplanned analyses did identify a target population for further evaluation of its potential as a DMOAD, and phase 3 trials are ongoing [130].

**Figure 2:**
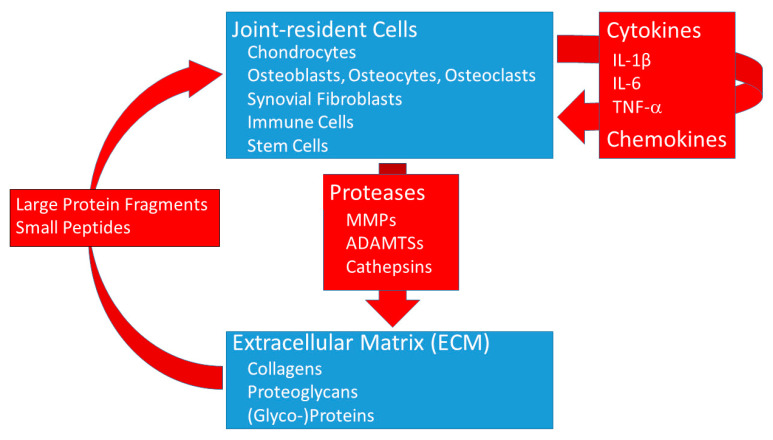
Proteases and cytokines in the joint. The different cell types present in the joint secrete proteases that degrade the complex cartilage extracellular matrix (ECM). This leads to a release of large protein fragments as well as the generation of small bioactive peptides. These degradation products can stimulate cells to produce more proteases and/or cytokines and chemokines that act either in an autocrine or paracrine manner and enhance the degeneration process in OA. In addition, cytokines and chemokines can influence the polarization of macrophages as well as the differentiation of stem cells. IL, interleukin; TNF, tumor necrosis factor; MMP, matrix metalloproteinase; ADAMTS, a disintegrin and metalloproteinase with thrombospondin motifs.

In summary, a better understanding of cartilage matrix degeneration, including identification of relevant proteases and cytokines as well as of bioactive cartilage fragments, is key to facilitate the development of novel, targeted treatment strategies (see Figure 2).

## 4. Impact of the Peripheral Nervous System and Its Neuropeptides on OA

### 4.1. Sensory Nerve Fibers in OA Joints

Soft tissues as well as bone are innervated by sensory nerve fibers, which are important for proper bone and joint development. In bone, sensory nerve fibers containing the neuropeptides substance P (SP) and alpha-calcitonin gene-related peptide (αCGRP) were found localized near blood vessels targeting the periosteum, bone marrow as well as compact and trabecular bone structures [131]. However, there is limited information available on sensory innervation and its changes in location and density in the OA-affected joint. Suri et al. localized sensory nerve fibers within vascular channels in mild and severe OA forms and reported that OA cartilage may be even innervated by a substantial number of fine nerve terminals [132]. The group describes the ingrowth of fine unmyelinated nerves into osteophytes through vascular channels originating mainly in subchondral bone. These nerves were perivascular localized and innervated the surface layer of articular cartilage, whereas free nerves, not associated with blood vessels, did not innervate the articular cartilage. Vascularization of the non-calcified cartilage matrix did occur also throughout early histological OA stages and was not restricted to end-stage OA. Another study revealed that the percentage of osteochondral channels containing CGRP positive nerves in symptomatic chondropathy was higher than that in asymptomatic chondropathy (difference: 2.5%) and additionally in meniscal transected rat knees compared to sham-operated knees (difference: 7.8%) [133]. These studies seem to imply that novel ingrowth of sensory nerve fibers occurs through the tidemark rather than coming from the synovium or periosteum. 

An in-depth study comparing the density of sympathetic and sensory nerve fibers between patients with arthrofibrosis (AF) and OA demonstrated that in OA knees, the synovial density of SP-positive sensory nerve fibers was significantly higher [134]. In AF compared to OA, the density of sensory nerve fibers was higher compared to the density of sympathetic nerve fibers in the region of the anterior recess and in the infrapatellar fat pad. This study indicates that AF patients suffer from hyperinnervation with sensory nerve fibers relative to sympathetic nerve fibers in the anterior compartments of the knee. SP is probably a critical factor in this process due to its profibrotic capacities, as it stimulates TGF-ß expression and, in that way, contributes to a higher degree of fibrotic scar formation in AF tissue. A study by the group of Kawarai, using the monosodium iodoacetate (MIA) model of OA induction in the hip of rats, showed that expression of CGRP outside of the joints in the ipsilateral L4 dorsal root ganglion (DRG) neurons was significantly higher in the MIA group than in the sham group from day 7 on post-induction [135]. This was confirmed by other groups [136,137], suggesting that CGRP is a marker of inflammatory pain. In this line, genome-wide association studies (GWAS) unbiasedly identified gene variants associated with knee pain, though the relationship between pain and OA phenotype is complex. Pain phenotypes such as “post-surgical pain”, “early stage OA pain” and “persistent OA” pain were supported by transcriptomic differences identifying distinct molecular pathways in the dorsal root ganglia of a mouse model of surgically induced OA [138]. Pathway analyses in the persistent pain phenotype suggested that the innate immune system and neuroinflammation are key pathways involved in pain pathology and, among other genes, nerve growth factor (NGF) is upregulated here. 

Reports, with respect to sensory nerve fiber density during OA pathogenesis in comparison to healthy joints, are controversial and the issue is not solved satisfactorily to date [10,139]. Usage of experimental OA mouse models mostly showed a dramatic decrease of sensory nerve fibers in synovial tissue [140,141]. Other studies of human OA reported either an increase in SP- and CGRP-positive nerve fibers in the synovial tissue of knee joints [142,143] or a decrease [144]. These are studies of the synovium in chronic OA and almost nothing is known between the pathological changes in the knee and changes in these nociceptive fibers innervation profiles during the acute early phase of the disease. Whether sensory nerve fibers are lost, remain unaltered, increase or change tissue distribution as a prerequisite of OA pathogenesis in humans remains to be determined. One can speculate that vascularization of cartilage is a critical factor in the process of the cartilage matrix breakdown in OA, as this facilitates the access of pro-inflammatory cytokines and proteases transported in the blood to the cartilage matrix. Together with neuropeptides, released from sensory nerves, this constellation contributes critically to the degradation of macromolecular matrix structures. However, in order to obtain deeper knowledge, it would require reproducible OA-animal models resembling the slowly progressing pathogenesis of human OA and enabling longitudinal studies from early onset of the disease to late stages. 

### 4.2. Sensory Neuropeptides in OA Joints

#### 4.2.1. Substance P Effects

**Figure 3:**
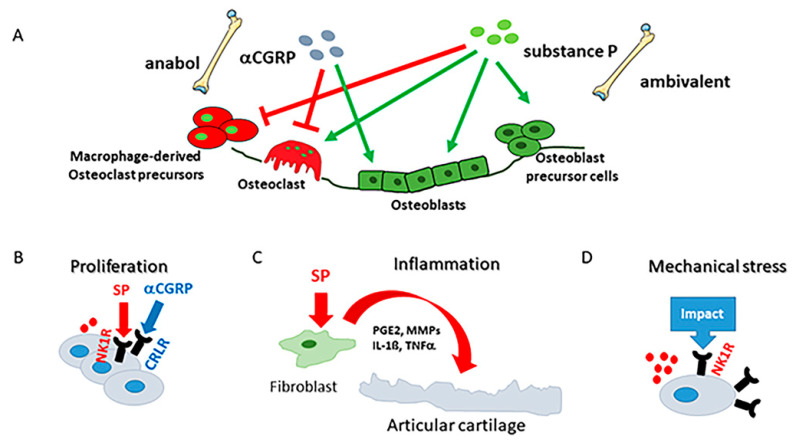
Sensory neuropeptides modulate bone and cartilage metabolism. (**A**) Effects of αCGRP (alpha-calcitonin gene-related peptide) and SP (substance P) on bone cell metabolism. Whereas αCGRP exerts mainly anabolic effects, SP signaling has an ambivalent nature. (**B**) Both substance P and αCGRP decrease proliferation of OA chondrocytes. (**C**) Stimulation of synovial fibroblasts with SP induces release of inflammatory mediators promoting cartilage degradation. (**D**) Application of mechanical load increases expression of NK1R (neurokinin 1 receptor) and endogenous synthesis of Substance P. CRLR, calcitonin receptor-like receptor; PGE2, prostaglandin E_2_. Adapted from reference [10].

Besides their classical function in nociception, SP and CGRP have extra functions in the musculoskeletal system, which can be summarized as trophic effects influencing the metabolism of the target cells (Figure 3A). SP can promote the proliferation, differentiation, apoptosis, matrix synthesis, and degradation of target cells through autocrine/paracrine modes (Figure 3B–D). Several studies demonstrated that SP modulates chondrocyte and bone cell function in a physiological setting and that chondrocytes and bone cells express its receptor neurokinin 1 (NK1R) (reviewed in [10,139,145]).

Contrary to the physiological situation, information on the effects of SP and its receptor in cartilage and subchondral bone in osteoarthritic conditions is sparse. Using a surgical murine OA model (DMM = destabilization of the medial meniscus), we did not find changes in NK1R expression or localization in meniscal sections as the proportion of receptor positive cells in relation to the total meniscal cell number was not affected by OA induction and/or neuropeptide absence [146]. However, in a recent study, it was tested whether genetic variations in the NK1R encoding gene (TACR1) are associated with pain level in individuals with radiographic knee OA [147]. Out of six single nucleotide polymorphisms (SNPs) in TACR1, one (rs11688000) showed a nominally significant association with a decreased risk of symptomatic OA versus asymptomatic OA in the cohort participants. The authors suggest that the TACR1 gene might contribute to the modulation of pain sensitivity in a subgroup of OA patients inheriting this SNP. 

The mechanical status of the joint is a central issue in OA when considering therapeutic approaches. For that, it is important to know that NK1R is involved in transduction of mechanical stress in chondrocytes (Figure 3D). It was demonstrated that SP is involved in mechanotransduction via the NK1R because the blockade of SP signaling by a chemical antagonist of the NK1R-inhibited chondrocyte responses to mechanical stimulation [148]. This group also demonstrated that normal and OA chondrocytes reacted differently to mechanical stimulation in that OA chondrocytes upregulated gene expression of the SP encoding gene, tachykinin (TAC) 1, whereas non-OA chondrocytes did not [149]. Challenging mice with DMM-induced OA, a model that is based on pathological alterations of mechanical loading in the knee joint, demonstrates that loss of SP accelerates sclerosis of the subchondral bone, being important already in very early OA bone pathology [146]. 

Absence of SP promotes heterotopic ossification of meniscal tissue, but leads to delayed cartilage degradation, implying the pleiotrophic effects of SP, depending on the tissue type. This study indicates both an anabolic and a catabolic effect of SP on bone homeostasis in OA, which was reported also from other groups. Xiao et al. observed an increased SP immunoreactivity in the cancellous bone of OA femoral heads compared to osteoporotic bone. Increased SP expression correlated positively with pain intensity, analyzed by the visual analog scale (VAS), but also with bone structural parameters demonstrated by µCT (micro-computed tomography) [150]. Concluding from this, SP might be implicated in causing OA pain, but also seems to preserve bone structure and may be the cause for a net positive balance of bone formation in OA pathophysiology. The molecular basis of how local release of SP in bone tissue contributes to bone metabolism is not known yet, but acting as a modulator of bone cell activity, SP could contribute to bone pathologies and SP targeted therapies could potentially target OA bone phenotypes, too. 

#### 4.2.2. αCGRP Effects

As for SP, not much is published about the effects and involvement of αCGRP in cartilage pathophysiology during OA progression. Recently, a study reported that the protein levels of αCGRP and iNOS (inducible nitrite monoxide synthase) were significantly upregulated in the dorsal rout ganglion (DRG) tissue of OA rats (induced via injection of MIA into knee joints) compared with the sham group at 6 weeks after model establishment. This suggests that the OA rats had neuronal damage and an increased inflammatory response in DRG tissue, resulting in a combination of inflammatory and neuropathic pain [151]. Very similar data were obtained in another study, where processed lipo-aspirate was injected into the rat joint after OA induction via MIA, leading to pain relief and anti-inflammatory responses [152]. Using the same OA model, another group observed a strong reduction of αCGRP-positive nerve fibers in the infrapatellar fat pad after treatment with platelet rich plasma (PRP) and associated this with the reduction of joint pain analysis of weight-bearing distribution with an incapacitance tester [153]. An increase in CGRP positive nerve fiber density was also observed in synovial tissue after MIA induced knee OA [154]. 

Of note, all these studies did not evaluate the possible trophic effects of αGCRP on joint tissues. This was done in a study by Nakasa et al., who detected increased αCGRP positive staining in the subchondral bone epiphysis after OA induction via DMM [155]. This group observed that under pathophysiological conditions, as in OA, inhibition of αCGRP effects by blocking its receptor with an antagonist, attenuated subchondral bone sclerosis. Consequently, articular cartilage erosion and degeneration was delayed in the early stage in this OA model. Of note, these effects were abrogated in a later stage of the disease (8 weeks after OA induction) indicating that other factors affect bone turnover compensating for αCGRP effects. These dual effects may also be attributed to αCGRP in pathological situations. In a recent study, it was suggested that αCGRP protects cartilage matrix integrity in murine OA caused by DMM as cartilage matrix stiffness is affected in the absence of αCGRP [146]. Cartilage matrix degradation according to OARSI score is not aggravated in the absence of αCGRP; however, it occurs earlier than in WT.

With respect to OA-associated subchondral bone alterations, it was shown that OA caused an upregulation of αCGRP in subchondral bone afferents over time, which displayed a strong correlation with the subchondral bone damage score [156]. An elegant study by Zhu et al. showed that an early increase in osteoclasts in an anterior cruciate ligament transection (ACLT) murine OA model was strongly related to an induction of CGRP-positive nerves in the subchondral bone. They found strong evidence that osteoclast derived netrin-1 promoted sensory nerve innervation which very likely is involved in mediating chronic OA pain [157].

Altogether, one can conclude that most reports of αCGRP effects in OA joints are related to increased pain sensation whereas only few reports analyzed trophic effects of αCGRP on bone cells or chondrocytes (Figure 3A,B). According to these observations, αCGRP seems to have cartilage protective effects but increased αCGRP release correlates with increased subchondral bone degradation in OA. A positive side effect of blocking αCGRP signaling is a reduction in subchondral bone sclerosis; however, subchondral bone sclerosis is increased in αCGRP-deficient mice after OA induction. These, at first glance, contradictory data suggest that αCGRP effects might be strongly time dependent, meaning that the whole life genetic ablation of the neuropeptide has different consequences than blocking its function only during OA pathogenesis. 

### 4.3. Neuropeptides as Biomarkers

As an important aspect in OA treatment, diagnosis and prognosis would be the identification of reliable biomarkers, which allow an early diagnosis before symptoms appear and before irreparable damage occurs in the joint tissues. The critical question here is, would sensory neuropeptide concentration in synovial fluid or serum (preferentially) change, and if so, could neuropeptides qualify as biomarkers for OA pathogenesis? Analysis of the concentration of both neuropeptides in the serum of wild type (WT) DMM and Sham mice 2 and 12 weeks after OA induction did not detect significant OA-related differences in SP and αCGRP serum concentrations [146]. By trend, SP concentration was reduced 2 weeks after DMM surgery and αCGRP concentration was, by trend, reduced over time from 2 to 12 weeks after surgery but was unaffected by OA induction. A prospective pre-clinical study including healthy dogs and dogs with unilateral lameness and joint pain in a single joint from naturally occurring OA analyzed the concentrations of 14 synovial OA biomarkers in synovial samples [158]. They detected a higher concentration of SP in the synovial fluid of OA joints, but no relationship was identified between biomarker concentrations and gait asymmetry in dogs with OA. 

The detection of higher levels of SP in synovial fluid from patients with RA and OA, and increased expression of NK1R, indicates possibly catabolic effects of SP on articular cartilage [159]. In other musculoskeletal diseases, such as developmental hip dysplasia, increased levels of SP and αCGRP detected in synovium and synovial fluid indicate also catabolic and pro-inflammatory effects of these neuropeptides [160]. αCGRP concentrations in human serum and synovial fluid correlate with increasing KL grade and are lowest in controls without OA diagnosis [161]. This means that higher concentrations of CGRP in serum and synovial fluid are indicative of worsening pain, more severe stiffness and poorer physical function, and are usually associated with more serious image presentations in knee OA patients. These observations indicated that concentrations/levels of CGRP in serum and synovial fluid might predict clinical severity for OA patients.

These studies imply that neuropeptides can be correlated with OA symptoms but might not be the most reliable biomarkers for OA diagnosis and prognosis. One reason may be the short half-life of SP and αCGRP in serum and synovial fluid, which would require stabilizing them during handling of the samples. However, deducing from these few studies, only αCGRP in serum and synovial fluid might predict clinical severity for OA patients.

OA is a complex disease, where every patient has little variations in cartilage, subchondral bone and neuronal phenotypes. This includes variations in distribution of SP- and CGRP-positive nerves in joint tissues, which might correlate with the severity of structural cartilage or subchondral bone alterations. Variations in synovial fluid/serum concentrations of these neuropeptides might additionally correlate with the individual joint radiographic status. 

Sensory nerve fibers and their neurotransmitters are crucial neuronal effectors regulating cartilage and bone physiology. Numerous resident cells of the osteoarticular system have receptors for sensory neurotransmitters and are able to respond to these stimuli. It becomes more and more evident that neuronal signaling critically influences tissue regeneration, i.e., after bone, osteochondral and meniscal traumata and tendon/ligament ruptures. A better understanding of changes in sensory neuropeptide supply in the context of increased pro-inflammatory and a lack of regenerative signaling factors might lead to novel strategies to halt OA progression and in improved therapies for the treatment of synovitis and cartilage and osteochondral lesions [10].

## 5. Conclusions and Open Questions

Recent advances in OA pathology have pointed to key roles of several new pathways, which can potentially be targeted with novel therapeutic strategies. Many clinical studies associated with OA have been conducted, which address mainly structural targets such as the articular cartilage and subchondral bone, often in combination with reduction in inflammation and nociceptive pain related to sensory joint innervation. In general, clinical success is marginal and so far, no treatment has been able to halt or reverse OA progression long term. Identification of selective disease time-points, preferentially in an early OA stage, and applying appropriate medication might help tailor individual treatment regimens for each patient subgroup in the future. The specific spatiotemporal pattern of early osteochondral OA changes seen in large animal models is in line with recent clinical investigations using advanced MRI technologies to quantify cartilage loss and/or gain in specific subregions. Since axial alignment and obesity are important contributors for OA progression, therapeutic approaches to redistribute loads across the joint, for example, by using insoles or performing high tibial osteotomy, or weight loss are recommended. 

A better understanding of the role of proteases and cytokines in OA has already resulted in novel treatment strategies targeting ADAMTS-5 activity, as well as Wnt, IL-1β- and FGF-18 signaling. Hopefully, future research in this area will identify more druggable molecular targets. 

Furthermore, modulation of derailed sensory nervous pathways might provide new opportunities not only to dampen pain but also to halt structural tissue deterioration. Importantly, it should be kept in mind that targeting one sensory neuropeptide might be counteracted by imbalanced expression of the other neuropeptide. However, as OA is a highly heterogeneous disease, a single therapeutic approach targeting a single joint tissue may not be effective. Diagnosis of early OA stages would be advantageous for the development of more efficient, targeted therapies. Identification of reliable biomarkers and more advanced imaging methods that are superior to radiographic diagnosis as well as stronger inter-disciplinary treatment regimens are indispensable. More basic science in reliable and clinically relevant translational (animal) disease models will help to better decipher the intricate mechanisms of OA and develop novel regenerative therapies. Personalized OA therapy is the ultimate goal, and recent advances in targeted drug development might provide a pool of suitable therapeutic options in the future. 

## Data Availability

Not applicable.
